# Radiological evaluation of a new straight electrode array compared to its precursors

**DOI:** 10.1007/s00405-020-06434-5

**Published:** 2020-10-22

**Authors:** Manuel Christoph Ketterer, A. Aschendorff, S. Arndt, I. Speck, A. K. Rauch, R. Beck, F. Hassepass

**Affiliations:** Department of Otorhinolaryngology-Head and Neck Surgery, Faculty of Medicine, Medical Center-University of Freiburg, University of Freiburg, Killianstrasse 5, 79106 Freiburg, Germany

**Keywords:** Cochlear implant, Electrode array, Dislocation, Scalar position, Cone beam computed tomography

## Abstract

**Objective:**

The aim of this study is to examine electrode array coverage, scalar position and dislocation rate in straight electrode arrays with special focus on a new electrode array with 26 mm in lengths.

**Study design:**

Retrospective study.

**Setting:**

Tertiary academic center.

**Patients:**

201 ears implanted between 2013 and 2019.

**Main outcome measures:**

We conducted a comparative analysis of patients implanted with lateral wall electrode arrays of different lengths (F24 = MED-EL Flex^24^, F26 = MED-EL Flex^26^, F28 = MED-EL Flex^28^ and F31.5 = MED-EL Flex^Soft^). Cone beam computed tomography was used to determine electrode array position (scala tympani (ST) versus scala vestibuli (SV), intracochlear dislocation, position of dislocation and insertion angle).

**Results:**

Study groups show no significant differences regarding cochlear size which excludes influences by cochlear morphology. As expected, the F24 showed significant shorter insertion angles compared to the longer electrode arrays. The F26 electrode array showed no signs of dislocation or SV insertion. The electrode array with the highest rate of ST dislocations was the F31.5 (26.3%). The electrode array with the highest rates of SV insertions was the F28 (5.75%). Most of the included electrode arrays dislocate between 320° and 360° (mean: 346.4°; range from 166° to 502°).

**Conclusion:**

The shorter F24 and the new straight electrode array F26 show less or no signs of scalar dislocation, neither for round window nor for cochleostomy insertion than the longer F28 and the F31.5 array. As expected, the cochlear coverage is increasing with length of the electrode array itself but with growing risk for scalar dislocation and with the highest rates of dislocation for the longest electrode array F31.5. Position of intracochlear dislocation is in the apical cochlear part in the included lateral wall electrode arrays.

## Introduction

All manufacturers diversify their electrode array portfolio more and more regarding shape, size, diameter and flexibility to enable the personalized choice of the implant. The relationship between cochlear morphology, electrode array position and postoperative speech discrimination is of increasing interest. Aschendorff et al. [2] first examined scalar position via rotational tomography for patients inserted with a Cochlear^®^ Contour (*n* = 21) versus a Cochlear^®^ Contour Advance^®^ (*n* = 22) electrode array (Cochlear Ltd., Lane Cove, Australia) and reported significantly higher speech discrimination results for scala tympani (ST) compared to scala vestibuli (SV) position. Further studies confirmed the beneficial initial ST position [13, 39]. Rotational tomography, cone beam computed tomography (CB-CT) and high resolution computed tomography (HR-CT) are widely accepted tools for the evaluation of the electrode array position detecting tip-fold over, scalar deviation or electrode misplacement (e.g. [1, 2, 9, 14, 24, 42]). The methods have been validated by histomorphological studies that included imaging and sectioning (e.g. [1, 6, 17, 23, 27]). Ketterer et al. [24] analyzed 403 ears in CB-CT inserted with a Cochlear^®^ Contour Advance^®^ electrode array (Cochlear Ltd., Lane Cove, Australia) and described that the electrode array was more likely to dislocate within cochleae with smaller height and smaller diameter. There is some evidence of less frequent dislocation rates in lateral wall (LW) arrays than in precurved arrays (e.g. [5, 6, 9, 36, 45]). Although, a newly developed slim precurved electrode array demonstrated 0% dislocation in both temporal bone studies and human implantation [1]. As already known for precurved electrode arrays, Wanna et al. [45] also stated that for LW electrode arrays an electrode position entirely within the ST leads to superior audiological outcomes.

Some studies described shorter electrode arrays (e.g. the Nucleus Hybrid L24 electrode array with 16 mm length) as being sufficient for hearing preservation, but as being insufficient for optimal speech perception production via electrical stimulation due to a less focused stimulation and increasing channel interaction [15, 22, 26, 38]. Atraumatic insertion does not only depend on surgical skills and electrode array design, but also on individual cochlear duct lengths, anatomical abnormalities, the angle of insertion determined by anatomical trajectory to the round window, as well as cochlear heights that determines the spiraling of the lumen [9]. Therefore, manufacturers produce electrode arrays with different designs and length to best suit individual anatomy. MED-EL (MED-EL GmbH Innsbruck, Austria) designed and produces LW electrode arrays of different lengths (20–31.5 mm).

Cochlear coverage of the electrode array and its influence on postoperative outcome have been discussed in many previous studies. Long arrays with 28 mm length or more can be inserted deeply and have therefore higher coverage rates. They might have the ability to stimulate not even the cochlear basal turn but also the cochlear apex. Previous and recent studies have reported that greater depth of insertion is associated with better audiological results [1, 7, 20, 21, 32, 34].

The aim of this study is to evaluate retrospectively the new LW electrode array (Flex^26^, MED-EL = F26) regarding scalar dislocation rate and electrode coverage compared to other LW electrode arrays with different electrode array lengths of the same manufacturer in correlation to cochlear size (Flex^24^ = F24, Flex^28^ = F28 and the Flex^Soft^ = F31.5; MED-EL G.m.b.H. Innsbruck, Austria). To the best of our knowledge, until now neither temporal bone nor human studies have been published to evaluate the new 26-mm long LW electrode array F26. This is the first study assessing the F26 electrode array regarding scalar position and dislocation behavior. Furthermore, to the best of our knowledge this is the first study evaluating the position of the dislocation in this type of LW electrode arrays.

## Material and methods

### Study and subject

We performed a retrospective analysis of adult patients implanted between 2013 and 2019 at the department of Otorhinolaryngology, Head and Neck surgery at the Implant Center of the University hospital Freiburg. HR-CT and magnetic resonance imaging (MRI) including contrast agent to exclude intrameatal or intralabyrinthine schwannoma have been conducted preoperatively. Patients with cochlear anomalies and signs of sclerosis were excluded of this study. Only patients inserted with a MED-EL Flex^24^ (F24), MED-EL Flex^26^ (F26), MED-EL Flex^28^ (F28) and MED-EL Flex^Soft^ (F31.5) electrode array were included in this investigation. Electrode arrays have been chosen by different criteria as cochlear morphology, surgical preference and in cases of residual hearing shorter arrays have been inserted. Patient´s sex, age, implanted side and cochlear size (distance *A* and *B* referring to Escudé et al. [11]) and product of the cochlear basal turn referring to Ketterer et al. [24] were analyzed. Partial inserted electrode arrays due to residual hearing have been excluded from this study resulting in a total of 6 patients (two patients with F28 and four with F31.5 electrode arrays) that have been excluded from further analysis.

### Radiological and morphological evaluation

**Fig. 1:**
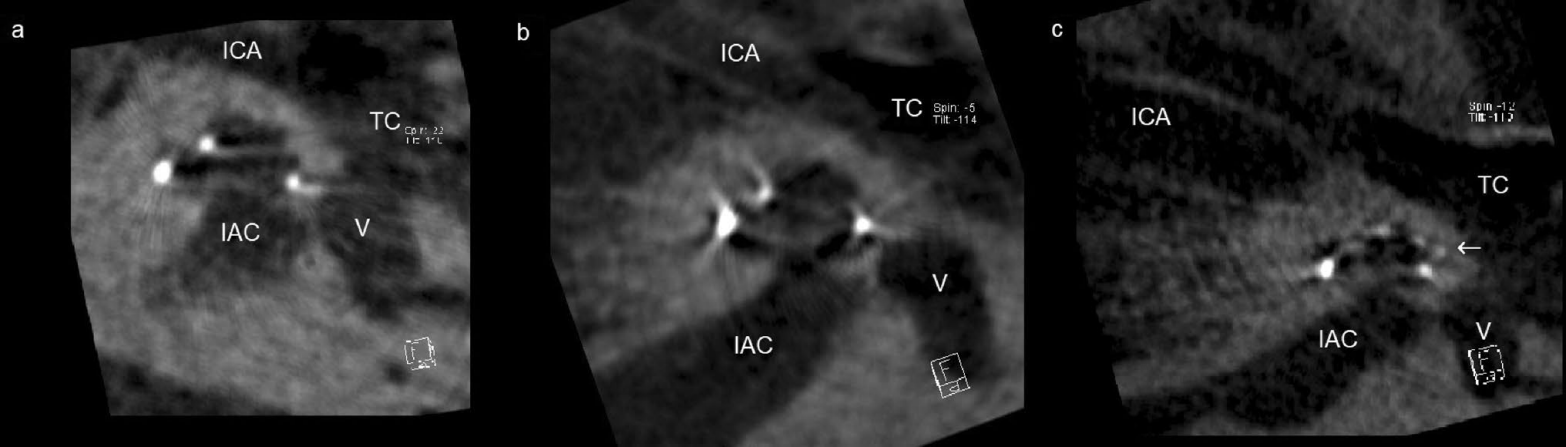
**a** CB-CT image of the Flex26 inserted in scala tympani without any signs of dislocation. **b** Flex28 inserted in scala vestibuli via cochleostomy. **c** FlexSoft inserted in scala tympani with a dislocation (arrow) to scala vestibuli. *ICA* internal carotid artery, *IAC* internal acoustic canal, *TC* tympanic cavity, *V *vestibulum)

We evaluated the scalar location of the electrode array postoperatively in all patients by CB-CT (DynaCT-equipped Axiom Artis dTA angiography unit; Siemens Co., Erlangen, Germany) [2, 3]. All included electrode arrays were fully inserted. Two physicians analyzed the scans regarding scalar electrode position (ST versus SV insertion, intracochlear dislocation, insertion angle) and cochlear size (diameters in length and width referring to Escudé et al. [11] see Fig. 1) independently, and used Impax 6 by Agfa Healthcare for reconstruction. The insertion angle has been evaluated between distance *A* and the bloom artefact of the apical electrode as described before by Ketterer et al. [24] (see Fig. 1).

### Statistics and ethics committee

We performed statistical analysis using Gnu R statistical computation and graphics system (ANOVA, Tukey’s Honest Significant Difference; GNU R, Version 3.0.3, Core Team, Vienna, Austria, https://www.R-project.org). We calculated our results descriptively and the level of significance was set at 5.0%.

This study was conducted in agreement with the University of Freiburg Ethics Committee according to the declaration of Helsinki (Washington, 2002) (Number of ethic committee approval: 406/19) and registered on German Clinical Trials Register (https://www.drks.de/DRKS00019807).

## Results

### Study cohort

**Fig. 2:**
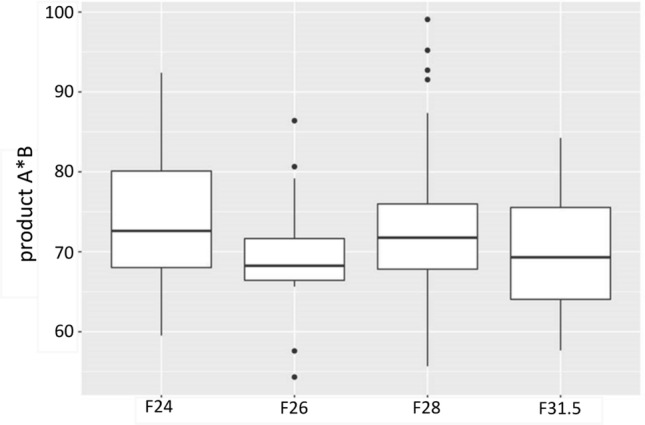
Distribution of the expanse of the cochlear basal turn (product of distance *A* and *B* [11] for each electrode array separately (*p* > 0.05). There is no significant difference regarding cochlear size between the included electrode array groups

Altogether we included 201 ears implanted between 2013 and 2019. We identified 99 left and 102 right cochleae. The mean age was 55 years. Tables 1, 2 and 3 shows the distribution of the study cohort. For analyzing electrode array design and position cochlear size must be included into the assessment to assemble the final insertion of the electrode arrays independently of cochlear size. No significant difference in cochlear size (distance *A* and *B* established by Escudé et al. [11] and cochlear basal turn product of distance *A* and distance *B* established by Ketterer et al. [24] see Fig. 2) was detected between our four defined electrode array groups, so that eventually there is no influence of the cochlear morphology on the described cochlear coverage.

**Table 1:** Distribution table of the study cohort and cochlear size measurements

	Mean	Standard deviation	Minimum	Maximum
Age (years)	55.0	16.4	18	83.4
Distance *A* (mm)	10.4	0.6	8.7	12.7
Distance *B* (mm)	6.9	0.4	5.6	8.1
Product *A* × *B *(mm^2^)	72.2	7.3	54.3	99.1

**Table 2:** Distribution of the included electrode arrays in total and percentage and their surgical management (cochleostomy versus round window insertion)

Distribution total *n* = 201	F24	*n* = 28 (13.8%)
	F26	*n* = 15 (7.5%)
	F28	*n* = 139 (69.2%)
	F31.5	*n* = 19 (9.5%)
Side	Left: 99	Right: 102
Inserted via	Round window: 110 in total (54.7%) F24: 8 (28.5%) F26: 4 (25%) F28: 64 (46%) F31.5: 15 (78.9%)	Cochleostomy: 91 in total (45.3%) F24: 20 (71.5%) F26: 11 (75%) F28: 75 (54%) F31.5: 4 (21.1%)

**Table 3:** Included electrode arrays (F24 = MED-EL Flex^24^, F26 = MED-EL Flex^26^, F28 = MED-EL Flex^28^ and F31.5 = MED-EL Flex^Soft^) and their dislocation behavior (T = scala tympani; TD = dislocation out of the scala tympani; V = scala vestibuli; VD = dislocation out of the scala vestibuli)

	T	TD	V	VD	Total
F24	27 (96.43%)	1 (3.57%)	0	0	28 (100%)
F26	15 (100%)	0	0	0	15 (100%)
F28	125 (89.93%)	6 (4.32%)	8 (5.75%)	0	139 (100%)
F31.5	13 (68.42%)	5 (26.32%)	0	1 (5.26%)	19 (100%)

### Cochlear coverage

**Fig. 3:**
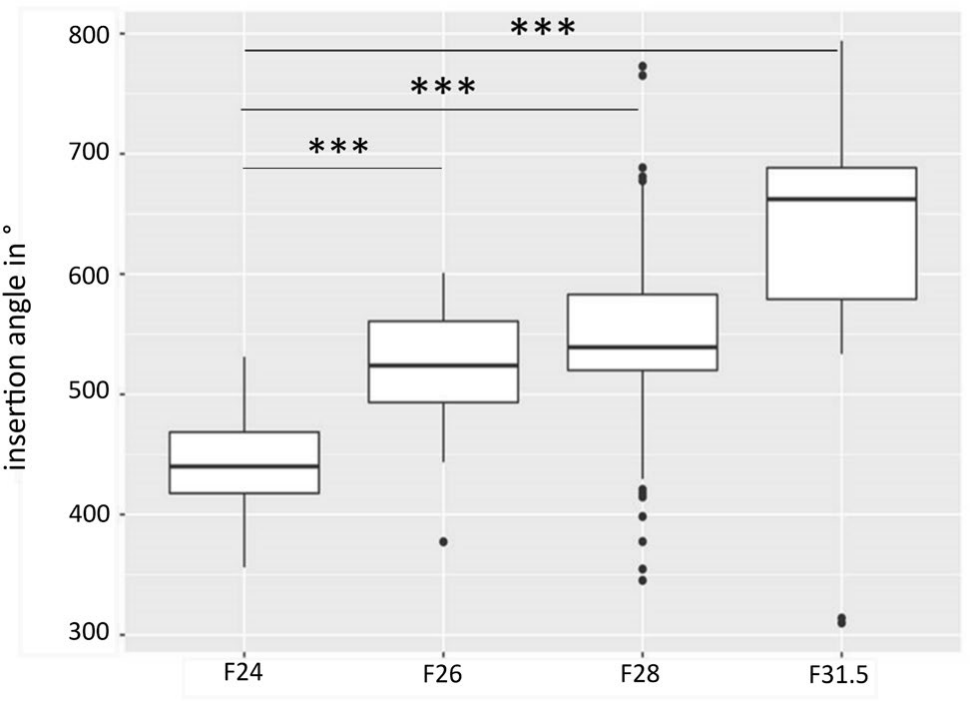
Insertion angle for each included electrode array. Statistical difference could have been found between all electrode arrays (all *p* < 0.008), except F26 versus F28 (*p* = 0.422)

Figure 3 shows the mean insertion angle for each included electrode array. As expected, within the study cohort the F24 showed significant different coverage compared to the longer electrode arrays F28 and F31.5 (*p* < 0.0001). Surprisingly we could not find significant different coverage between the F26 and F28 group (*p* = 0.42) (see Table 4: electrode array coverage). As mentioned before, the cochlear size comparing the F26 and F28 group was not significantly different.

**Table 4:** Electrode array coverage (in °)

	Mean	Standard deviation	Minimum	Maximum
F24	443.3	43.9	356.1	531.2
F26	517.0	60.36	377.4	601.0
F28	546.8	68.0	354.3	772.8
F31.5	616.7	124.4	309.9	794.1

### Dislocation rates and the position of dislocation

**Fig. 4:**
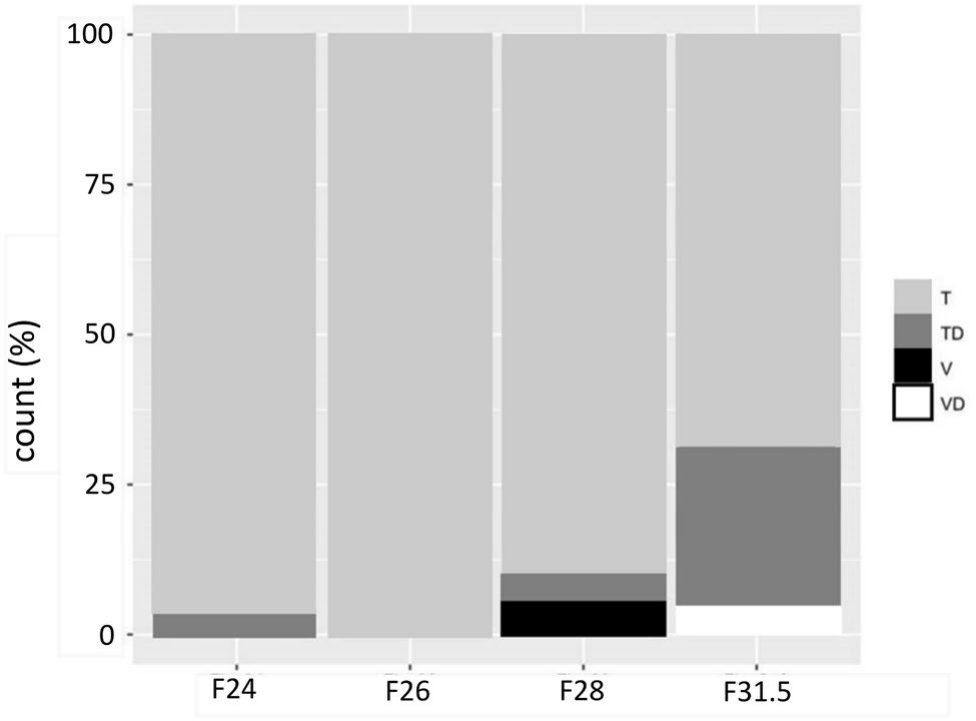
Electrode array position for each included electrode array (T = scala tympani; TD = tympani dislocation; V = scala vestibuli; VD = vestibuli dislocation) (see also Table 3/counts, total and percentages)

**Fig. 5:**
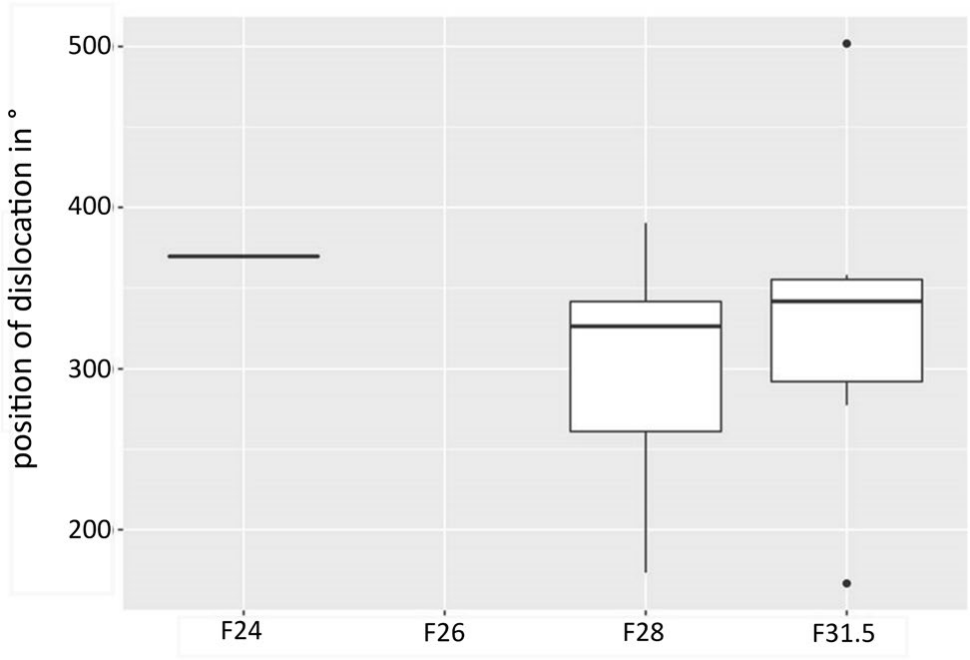
Position of intracochlear electrode array dislocation in ° for each included electrode array. The F26 showed no dislocations (mean total: 346.4°; mean F24: 360°; mean F28: 335°; mean F31.5: 344°)

No scalar dislocation or SV insertion was present for the new LW electrode array F26 (Table 3). The electrode array with the highest rate of scalar dislocations (27.78%) was the F31.5. The F28 showed 4.58% dislocated electrode arrays and the F24 showed only one dislocation (3.57%) (see Fig. 4). Both, round window and cochleostomy insertions have been performed in all electrode array specific subgroups (see Table 2). Comparing the study cohorts of cochleostomy versus round window inserted electrode arrays we could find 10 dislocations (F24; F28; F31.5) and 8 SV insertions (F28) for cochleostomy inserted electrode arrays (Table 3). For round window insertions a dislocation from ST to SV was detected in two cases (F28 and F31.5). In all cases of round window insertions, we detected a primary ST insertion. Most of the LW electrode arrays in the present study dislocated between 320° and 360° (see Fig. 5).

## Discussion

### Study cohort and cochlear coverage

Cochlear implantation focuses on reducing trauma during insertion to preserve residual hearing and prevent scaring. Appropriate cochlear electrode array design including electrode array length resulting in less traumatic surgical techniques is particularly important in this respect [10]. To the best of our knowledge, this is the first study evaluating the scalar position of the new LW F26 electrode array by MED-EL. Furthermore, we detected the specific position of dislocation in LW electrode arrays in the largest study cohort evaluated so far with 201 implanted ears. In the present study, cochlear sizes of all four electrode array design groups did not show significant differences so that a direct comparison of the four groups is ensured. Previous literature described cochlear coverage in average- sized models [8] and reported that angular insertion depth depends on both electrode array length and cochlear size. Ketterer et al. [24] demonstrated significant differences in human cochlear morphology. Though, electrode array comparisons are valid, because the examined electrode array groups included in this study do not differ regarding cochlear size.

### Dislocation rates

This study demonstrates significant different dislocation behavior of the included LW electrode array designs. The shorter F24 and the new straight electrode array F26 show less (F24: *n* = 1) or no signs (F26) of scalar dislocation or SV insertion, neither for round window inserted nor via cochleostomy inserted electrode arrays. Nordfalk et al. [30] and O’Connell et al. [33] also found no scalar dislocation of the F24. Nevertheless, the cohorts of the aforementioned studies [30, 33] were considerably smaller.

The electrode array with the highest rate of dislocations in our study was the F31.5 electrode array (26.3%), which is the longest array in the present study. Most of the studies that examined the F31.5, did not find and describe dislocations for this certain array ([5], F31.5 *n* = 9) or did not analyze their data for dislocations at all ([4], F31.5 *n* = 8). Comparing the published CB-CT images and the used flat panel detector of Boyer et al., the CB-CT used in this study provides higher resolution and we also included 19 instead of 9 F31.5 inserted patients [5].

Recent reviews [8, 9] of 26 articles, described the incidence rate of scalar dislocations for 21 different electrode arrays of five different manufacturers (Advanced Bionics, Valencia, CA, USA, Cochlear Ltd., Lane Cove, Australia; MED-EL GmbH Innsbruck, Austria; Advanced; Oticon Inc., Somerset, NJ and Nurotron Biotechnology Co. Ltd. Hangzhou, China). While a total of 424 ears implanted with precurved electrode arrays showed scalar dislocation (incidence rate: 32%), LW electrode arrays accounted to a total number of scalar dislocation rate of only 6.7% (34/507) [9]. This data is in line with the results for LW electrode arrays of our study (this study: 5.97% of ST dislocations in the total study cohort/see Table 3).

Regarding the reported 21.6% dislocation rates of the Cochlear^®^ Contour Advance^®^ electrode array by Ketterer et al. [24], the straight F31.5 (dislocation rate: 26.3%) in the present study and the precurved Contour Advance^®^ electrode array are the electrode arrays used and analyzed nowadays with the highest rate of dislocations. Both have wider basal diameter and are more rigid than shorter electrode arrays designed within the last years. Aschendorff et al. [1] described that all patients (*n* = 44) implanted with the new slim precurved electrode array (CI 532) of Cochlear ™ exhibited a complete ST insertion without dislocation in round window and cochleostomy approaches. Nevertheless, surgeons shall be careful with over insertion and tip-fold overs [1]. Therefore, we hypothesize that in LW as well as in precurved electrode arrays slim and more flexible electrode array design significantly reduces cochlear trauma and scalar dislocation.

The design of the electrode array and the influence of scalar position and dislocation on preserving residual hearing are still disputed. Nordfalk et al. [31] found a loss of residual hearing in patients with traumatic intracochlear dislocation using the PTA method of Helbig et al. [18]. Nevertheless, they did not have the statistical power to show significances and included only 13 patients with five different electrode arrays. Previous studies showed that the success of preserving residual hearing depends on intracochlear damage [25]. Soda-Merhy et al. [40] compared straight and perimodiolar electrode arrays at residual hearing rates across frequencies and described no significant difference. Nonetheless, some studies reported a higher loss of residual hearing in straight electrode arrays with increasing angular insertion depth [33]. Furthermore, the influence of the insertion technique comparing round window versus cochleostomy on residual hearing is still part of CI research discussion. Even though, Hassepass et al. [16] described no significant difference for the insertion technique evaluating the straight electrode array of a different brand (Cochlear™).

This study is the first study showing that straight electrode arrays dislocate at approximately 360° within the second cochlear turn, whereas studies published before [5] described the position of dislocation in perimodiolar arrays at 180° within the cochlear basal turn. Therefore, further prospective studies are necessary to evaluate the influence of cochlear dislocation within the second turn in straight arrays inserted via round window versus cochleostomy on the preservation of residual hearing.

### The position of dislocation

Most of the included LW electrode arrays in the present study dislocated between 320° and 360°. Boyer et al. [5] analyzed 61 CB-CT scans of 54 patients (31 ears with a perimodiolar versus 30 ears with a LW electrode array) and reported of eight perimodiolar electrode arrays with a dislocation from ST to SV. The different LW electrode array designs evaluated in their study (MED-EL F24, F28, F31.5 (= Flex^Soft)^ and Flex^Standard^), showed only one dislocation from ST to SV, which was one from the longest LW electrode array design cohort (Flex^Standard^ length: 31.5 mm) [5]. The Flex^Standard^ and F31.5 both have the same length of 31.5 mm but differ in design and flexibility. Boyer et al. [5] speculated that with precurved arrays, dislocation usually occurs in the ascending part of the basal turn of the cochlea [5]. The LW electrode array dislocated at approximately 370° in their study, whereas perimodiolar electrode arrays dislocated at around 170°–190° [5]. Our investigation confirms their assumption (see Fig. 4) and extends the previous knowledge of LW electrode array coverage and dislocation behavior with a higher number of implanted arrays. Since the cochlear sizes of the presented electrode array groups are comparable, we assume that the position of dislocation is design specific in LW electrode arrays of the included manufacturer. In this respect, the surgeon should keep this in mind during insertion to prevent dislocations. For future surgeries, we recommend measuring cochlear size in diameter preoperatively to choose the best fitting electrode array primarily in terms of cochlear size even if residual hearing, preference of the patient, anatomy and underlying medical (ear) conditions influence the decision. Instruments like the Otoplan© planning software by MED-EL have been established in the last years to assist the surgeon in measuring the cochlear and to find the best fitting array. These measurements and demonstration of the results to the patient can also help to bring the issue to the attention of the patient prior to CI surgery.

### Limitations of the study

A limitation of this study is that the F26 inserted patients recently got implanted and therefore reliable and comparable postoperative speech discrimination results are pending. Further studies should evaluate the influence on long-term postoperative speech perception and hearing preservation. A follow-up comparison will extend the knowledge about the F26 and its dislocation behavior by outcome results.

## Conclusion

This is one of the first studies evaluating the new straight electrode array F26 which shows no signs of scalar dislocation and intracochlear trauma, neither for round window nor for cochleostomy inserted electrode arrays compared to is precursors. The most frequent scalar dislocations occur in the longest electrode array designs. Scalar dislocations in LW electrode arrays occur at a predetermined angle at approximately 320°–360°.
